# Optimizing protocols for extraction of bacteriophages prior to metagenomic analyses of phage communities in the human gut

**DOI:** 10.1186/s40168-015-0131-4

**Published:** 2015-11-17

**Authors:** Josué L. Castro-Mejía, Musemma K. Muhammed, Witold Kot, Horst Neve, Charles M. A. P. Franz, Lars H. Hansen, Finn K. Vogensen, Dennis S. Nielsen

**Affiliations:** Department of Food Science, Faculty of Science, University of Copenhagen, Rolighedsvej 26, Frederiksberg, Denmark; Department of Biology, Faculty of Science, University of Copenhagen, Universitetsparken 15, Copenhagen, Denmark; Department of Microbiology and Biotechnology, Max Rubner-Institut, Kiel, Germany; Department of Environmental Science, Aarhus University, Frederiksborgvej 399, Roskilde, Denmark

**Keywords:** Quantification of bacteriophages, Extraction procedures, Feces, Gut microbiome, Phage metavirome

## Abstract

**Background:**

The human gut is densely populated with archaea, eukaryotes, bacteria, and their viruses, such as bacteriophages. Advances in high-throughput sequencing (HTS) as well as bioinformatics have opened new opportunities for characterizing the viral communities harbored in our gut. However, limited attention has been given to the efficiency of protocols dealing with extraction of phages from fecal communities prior to HTS and their impact on the metagenomic dataset.

**Results:**

We describe two optimized methods for extraction of phages from fecal samples based on tangential-flow filtration (TFF) and polyethylene glycol precipitation (PEG) approaches using an adapted method from a published protocol as control (literature-adapted protocol (LIT)). To quantify phage recovery, samples were spiked with low numbers of c2, ϕ29, and T4 phages (representatives of the *Siphoviridae*, *Podoviridae*, and *Myoviridae* families, respectively) and their concentration (plaque-forming units) followed at every step during the extraction procedure. Compared with LIT, TFF and PEG had higher recovery of all spiked phages, yielding up to 16 times more phage particles (PPs) and up to 68 times more phage DNA per volume, increasing thus the chances of extracting low abundant phages. TFF- and PEG-derived metaviromes showed 10 % increase in relative abundance of *Caudovirales* and unclassified phages infecting gut-associated bacteria (>92 % for TFF and PEG, 82.4 % for LIT). Our methods obtained lower relative abundance of the *Myoviridae* family (<16 %) as compared to the reference protocol (22 %). This decline, however, was not considered a true loss of *Myoviridae* phages but rather a greater level of extraction of *Siphoviridae* phages (TFF and PEG >32.5 %, LIT 22.6 %), which was achieved with the enhanced conditions of our procedures (e.g., reduced filter clogging). A high degree of phage diversity in samples extracted using TFF and PEG was documented by transmission electron microscopy.

**Conclusions:**

Two procedures (TFF and PEG) for extraction of bacteriophages from fecal samples were optimized using a set of spiked bacteriophages as process control. These protocols are highly efficient tools for extraction and purification of PPs prior to HTS in phage-metavirome studies. Our methods can be easily modified, being thus applicable and adjustable for in principle any solid environmental material in dissolution.

**Electronic supplementary material:**

The online version of this article (doi:10.1186/s40168-015-0131-4) contains supplementary material, which is available to authorized users.

## Background

The human gut hosts trillions of microbial cells belonging to all three domains of life, but among them, bacteria are the microorganisms dominating this highly competitive environment [1]. During the last decade, characterization of the gut microbiome (GM) has received extensive interest driven by a number of novel findings showing the influence of GM on human health and disease [2–4]. The focus, however, has mainly been oriented to characterize the prokaryote members of the GM, while efforts for characterizing the diversity and structure of bacteriophage communities have so far been relatively limited.

Bacteriophages (phages) are the most abundant biological entities found on earth [5], and their importance has been highlighted in many habitats such as aquatic environments, soil, food manufacturing environments, and the gastrointestinal tract [6–9]. A number of recent publications have reported the diversity of bacteriophages within GM [9–11], the factors driving their dynamics [1, 12, 13], their influence on the GM structure [14–16], and their association with dysbiosis and human disease [17–19]. Recent advances in high-throughput sequencing (HTS) technologies have opened new opportunities for exploring phage diversity, as well as their evolution and influence on the structure of bacterial communities in the human gut. However, despite this, little attention has been given to the efficiency and optimization of current protocols dealing with extraction of fecal bacteriophages prior to metavirome studies.

Current protocols employed for extraction of viral particles from fecal and environmental samples incorporate variations over the following steps: (i) dissolution, (ii) centrifugation, (iii) sequential filtrations (either dead-end or tangential) used first for removing cells and then for concentrating viruses, and (iv) final purification by CsCl gradient ultracentrifugation [20–25]. In spite of this shared backbone, protocols differ from study to study, as different centrifuge speeds, filter cut-off values, and concentration techniques are being used. Recently, Hoyles et al. [26] reported that through the inclusion of 0.45-μm filters, instead of 0.22-μm cut-off filters, it is possible to double the yields of viral DNA isolated from human feces and cecal fluids. However, determining the efficiency of extraction of viral particles from fecal samples by (i) spiking with known phages, (ii) measuring the impact of extraction procedures on DNA purity (presence of bacterial and host derived DNA) for mining auxiliary metabolic genes, and (iii) profiling the metagenomic virome as measurements of true success have not been performed to date.

Different kits for extraction of DNA from fecal samples have been shown to influence community structure as determined by HTS [27, 28]. Further, it is well known that viruses differ in size and structure, so differences in extraction procedures are likely to influence the structure of the phage community and amount and quality of extracted DNA and further metagenomic analyses, which in turn could lead to inaccurate or biased conclusions [29]. Further, it is important to point out that obtaining higher enrichments of viral particles will not only improve the yields of DNA but also give more accurate estimations of their concentration in stool samples (and the human gut). In addition, high phage yield will increase the probability of retaining relatively rare phages that otherwise would not be represented in the metagenomic dataset [30].

Here, we report two optimized protocols for extraction of bacteriophages from fecal samples prior to phage-metavirome studies. The optimization of protocols was based on two well-known laboratory techniques, tangential-flow filtration (TFF), and polyethylene glycol precipitation (PEG), using an adapted method from a published protocol [20] that has been used in several previously published studies [1, 9, 31] as control (literature-adapted protocol (LIT)). We analyzed the recovery rate of the three methods for a set of spiked bacteriophages (c2, ϕ29, and T4; representatives of the *Siphoviridae*, *Podoviridae*, and *Myoviridae* families, respectively), their efficiency to concentrate phage particles (PPs), and their impact on the phage community structure through metagenomic analyses. Our optimized methods not only yielded a higher number of PPs and DNA compared to the control but also demonstrated a higher efficiency for recovering spiked bacteriophages and increased the proportion of phage-derived sequences.

## Results

### Strategy for optimization of protocols

**Fig. 1:**
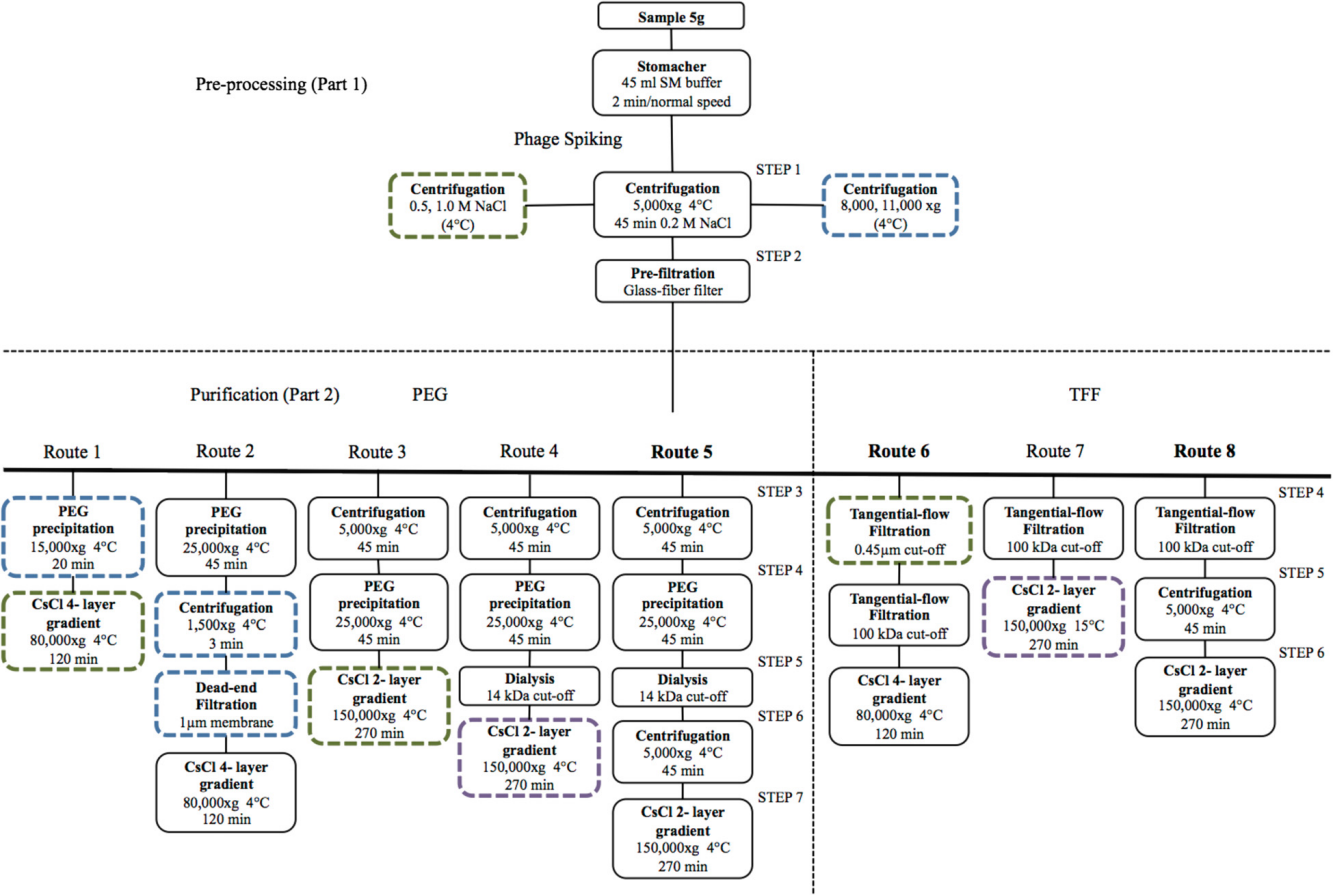
Strategy for optimization of phage extractions protocols. Pre-processing (part 1) was used to remove and sediment large particles; purification (part 2) aimed to remove low molecular weight impurities and microbial cells. *Boxes drawn with non-continuous lines* represent steps that were not suitable for extraction of bacteriophages (heavy loss of spiked phages). *Green-line boxes* depict filthy or impure samples (particularly low molecular weight impurities, assessed by visual inspection and TEM). *Blue-line boxes* show pathways where ≥50 % of the population of spiked bacteriophages was lost. *Purple-line boxes* indicate presence of microbial cells. The optimization of procedures for purification started in routes 1 (for the PEG approach) and 6 (for the TFF approach). Throughout this optimization, the routes were diverged into new extraction routes (i.e., from route 1 to route 2) until the highest recovery rate of the spiked bacteriophages was reached. Route 6 represented the LIT protocol. Base on their efficiency, route 5 (PEG) and route 8 (TFF) became the optimized protocols

The strategy for the optimization of protocols is illustrated in Fig. 1 and relied basically on minimizing the loss of known (spiked) bacteriophages at each step of extraction. Several approaches including different concentrations of NaCl and speeds of centrifugation/ultracentrifugation, dead-end and tangential-flow filtrations, two PPs concentration techniques (PEG and TFF), as well as four- and two-layer CsCl gradients (Fig. 1) were investigated. For convenience, the procedures for extraction were divided in two sections: the pre-processing (or part 1) was used to homogenize and remove large particles, whereas the purification section (or part 2) aimed at eliminating low molecular weight particles (impurities) and microbial cells and to concentrate phages. Representative populations of the *Siphoviridae*, *Podoviridae*, and *Myoviridae* bacteriophage families (a set of c2, ϕ29, and T4 phages) were inoculated to a concentration of 5.0 × 10^5^ phages per sample (5 g of feces re-suspended in 45 ml of SM buffer). If losses of more than 50 % of the spiked phages were observed in a given step of extraction (data not shown), these were consequently diverged into a new extraction route until a higher recovery of the inoculated populations was reached (free of impurities and contaminating cells). Instead of applying a 0.22-μm cut-off for tangential-flow filtration as described in previous studies [9, 12, 20], the reference (LIT) method was coupled with a 0.45-μm membrane cut-off as recently suggested [26]. The routes with the highest phage recovery, which eventually became the optimized protocols (PEG, route 5; TFF; route 8), were compared to the reference method (LIT, route 6) [20] based on their concentration of PPs (in the final phage concentrate collected from CsCl gradients) and yields of DNA. Each of the optimized routes of pre-processing and purification were assessed with fecal samples from three subjects and using two biological replicates as shown in Additional file 1.

### Recovery of spiked phage-representatives

**Fig. 2:**
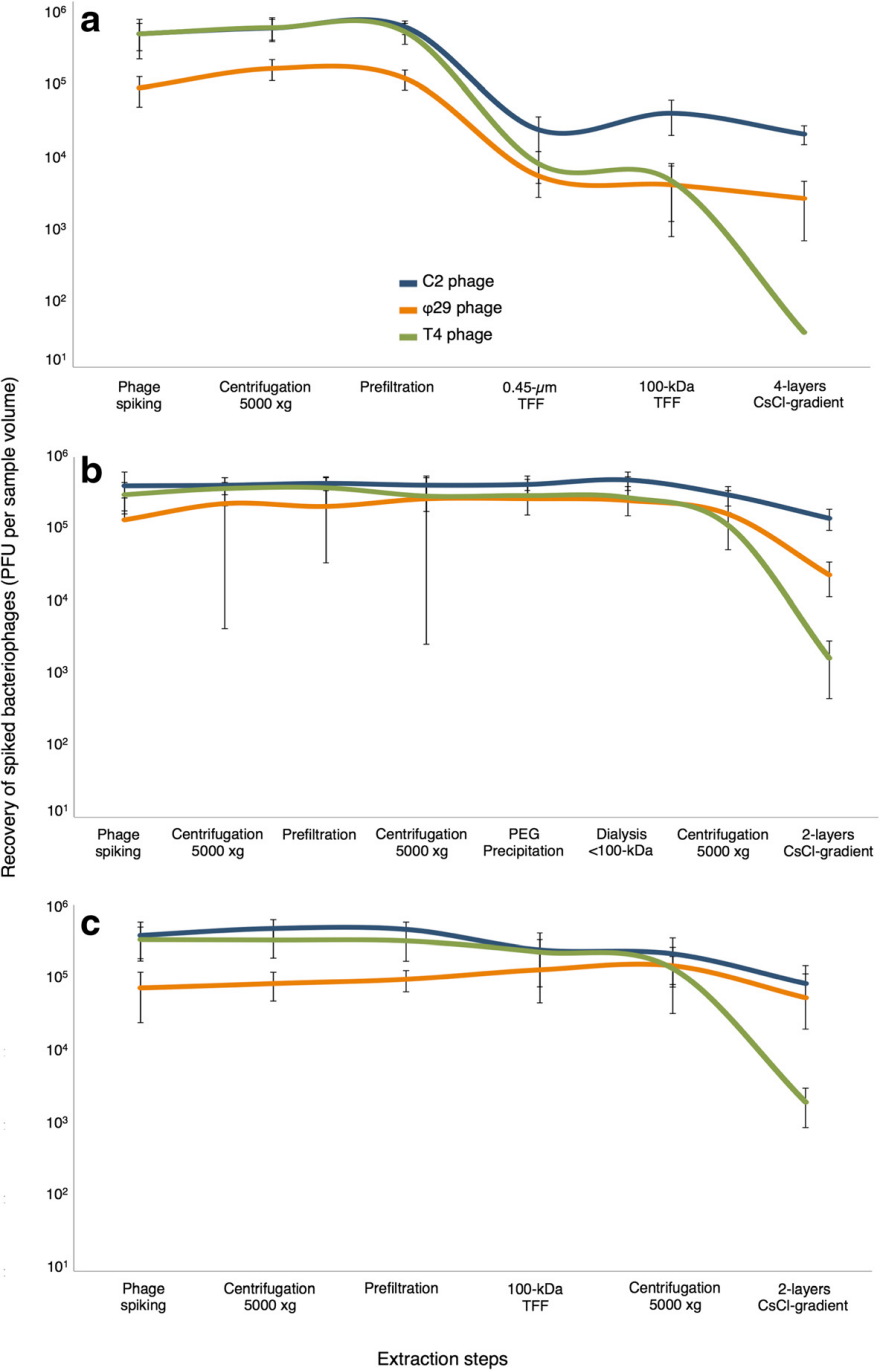
Bacteriophage recovery monitored in LIT (**a**), PEG (**b**), and TFF (**c**) protocols. The fecal samples were spiked with a set of bacteriophages, c2, ϕ29, and T4, and used as a process control for protocol optimization. The population of bacteriophages was monitored at each step by plaque assay (plaque-forming units (PFU)). Reductions in the curves of phage population indicate the degree of loss at a given step

Throughout the entire extraction procedure, PEG and TFF (Fig. 2b, c) showed an enhanced efficiency by retrieving greater phage populations than the control method (Fig. 2a). Compared with LIT, the optimized procedures were able to recover 8–13 times, 13–36 times, and 286–324 times larger phage populations of c2, ϕ29, and T4, respectively. Using the TFF and PEG protocols, virtually no spiked phages were lost until the ultracentrifugation step (CsCl gradient), whereas the literature-adapted protocol (Fig. 2a) lost up to 90 % of the spiked phages at the tangential-flow filtration step using a 0.45-μm membrane cut-off filter. The infectivity of the spiked T4 phage was compromised during the ultracentrifugation step (CsCl gradients), which occurred independently from the two speeds of ultracentrifugation used (using 82,000 and 150,000×*g*, for the control and optimized procedures, respectively) and possibly due to tail alterations at this step. Nevertheless, by performing a T4-specific qPCR analysis [32], we observed that T4 DNA was equally detected in samples that were treated with and without DNAse I after ultracentrifugation and prior to DNA extraction (data not shown), thereby suggesting that in spite of the infectivity loss, the phage head remained undamaged and therefore retained the genetic material.

### Quantification of PPs and yields of phage DNA

**Fig. 3:**
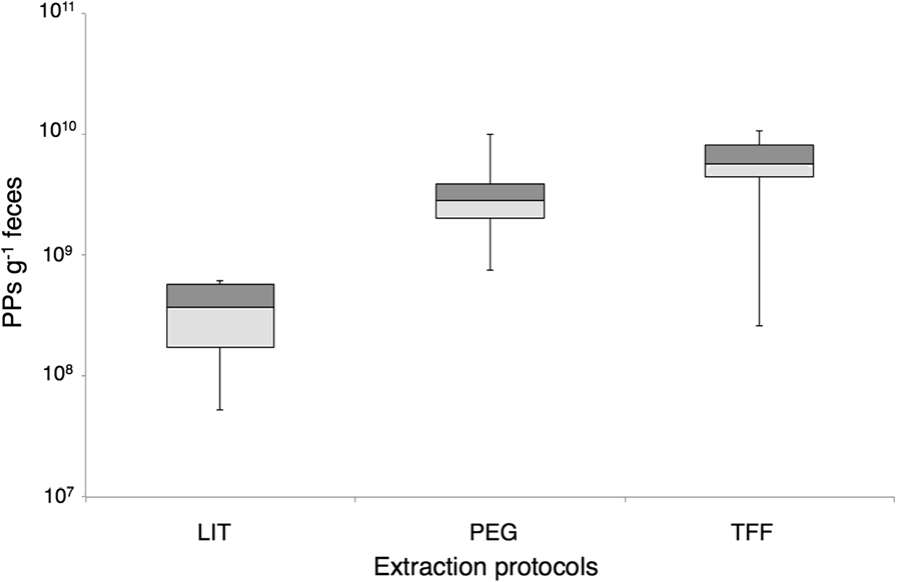
Quantification of phage particles (PPs) extracted with the adapted and optimized protocols. PPs were quantified in the final viral concentrate (CsCl fractions) by epifluorescence microscopy. *Values* shown represent the measurements of three fecal samples using two biological replicates

As determined by epifluorescence microscopy [20, 33], the abundance of phages determined per gram of wet material in samples extracted with LIT varied from 5.2 × 10^7^ to 6.2 × 10^8^ PPs, whereas for PEG and TFF the abundance ranged from 7.5 × 10^8^ to 1.0 × 10^10^ and 2.6 × 10^8^ to 1.1 × 10^10^ PPs, respectively (Fig. 3). A high variability in the number of PPs quantified within each extraction method was seen, but this was primarily an outcome of high interpersonal (fecal sample to fecal sample) variation. On average, the number of PPs extracted with LIT represented only 6 and 10 % of the PPs yields obtained with TFF and PEG, respectively. Furthermore, DNA yields (determined by fluorometry) obtained with LIT varied from 0.2 to 3.2 ng of DNA per gram of fecal material (Additional file 2) and resembled concentrations previously obtained [20] from 1 g of feces (~1–6 ng of DNA). In our optimized procedures, the quantity of DNA obtained ranged from 36 to 170 ng g^−1^ and from 13 to 58 ng g^−1^ in samples extracted with TFF and PEG, respectively.

### Characterization of PPs by transmission electron microscopy

**Fig. 4:**
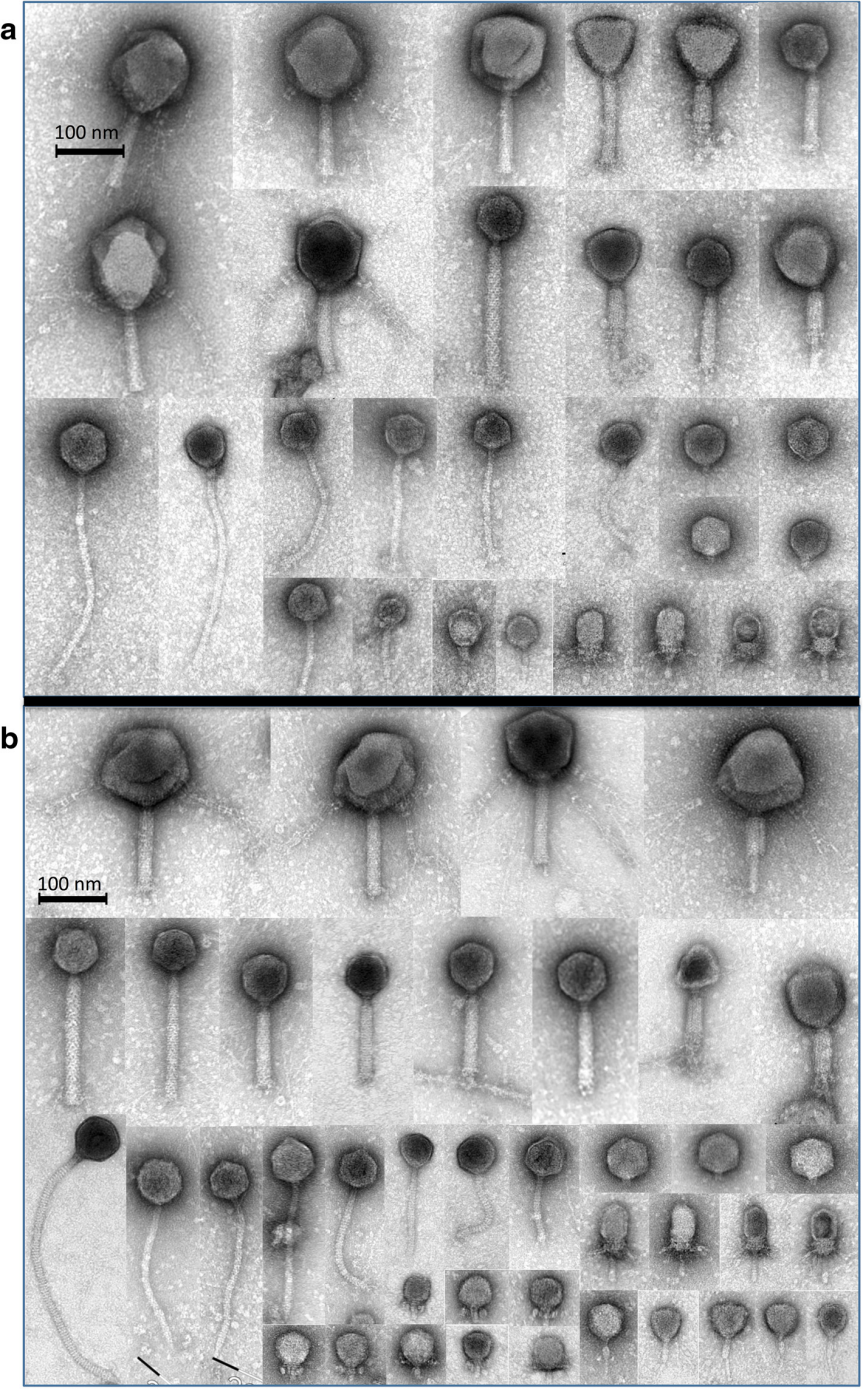
Electron micrographs of representative gut phages from subject X1. Phages extracted with TFF (**a** tangential-flow filtration, X1-TFF) and PEG (**b** polyethylene glycol precipitation, X1-PEG) procedures

**Fig. 5:**
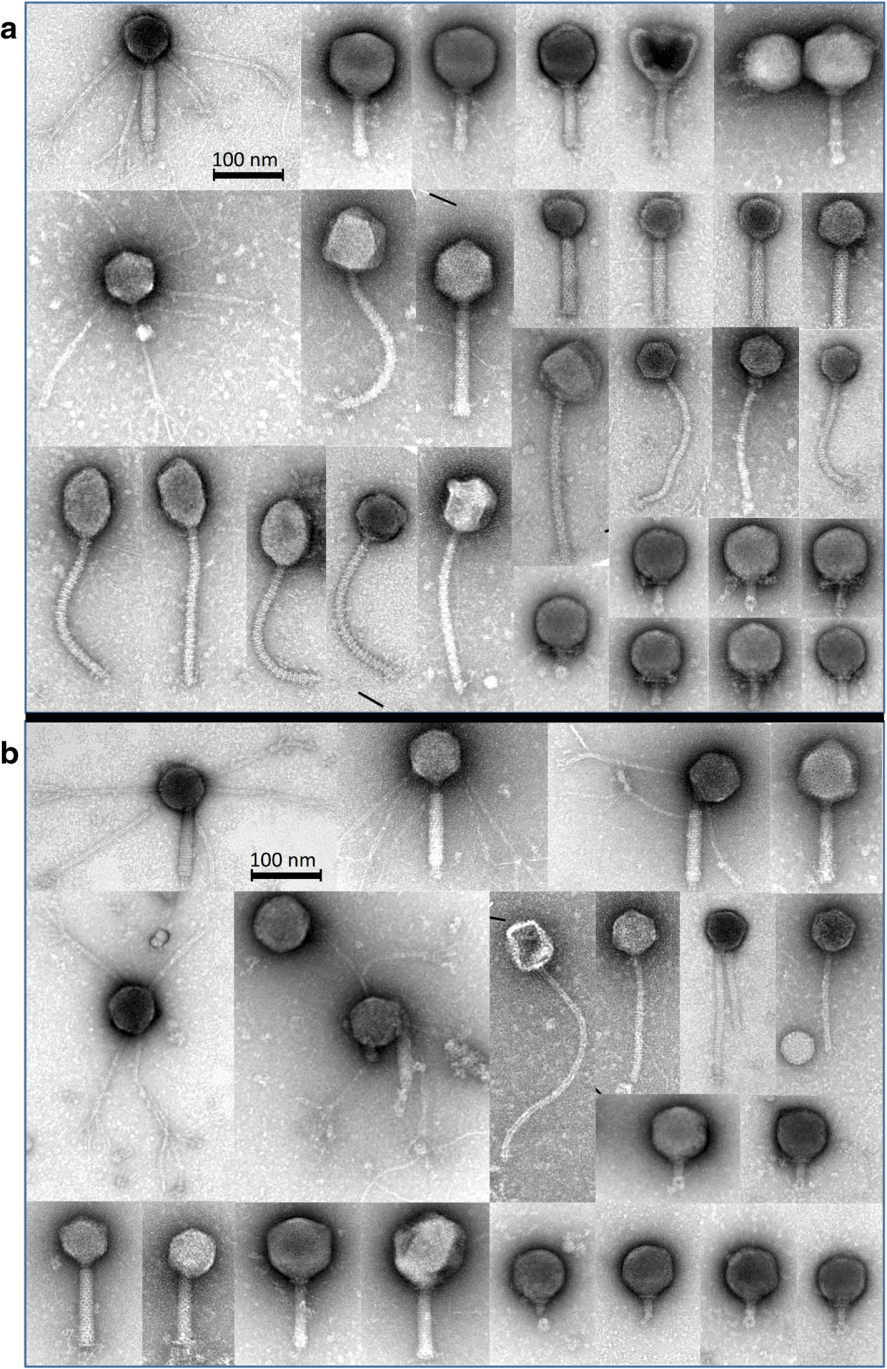
Electron micrographs of representative gut phages from subject Y1. Phages extracted with TFF (**a** tangential-flow filtration, Y1-TFF) and PEG (**b** polyethylene glycol precipitation, Y1-PEG) procedures

Phage characterization by transmission electron microscopy (TEM) [26] was carried out in samples collected from two subjects (samples X1-LIT, X1-PEG, X1-TFF, Y1-LIT, Y1-PEG, and Y1-TFF, see Additional file 1). For Y1-LIT and X1-LIT, no phage particles were observed by TEM as the low yield of PPs extracted were below the detection limit. For X1, the number of PPs extracted with our procedures was estimated to be 10^8^ PPs g^−1^ of wet material, and various *Myoviridae* (including *Peduovirinae*) *Siphoviridae* and *Podoviridae* and putative unclassified phage morphotypes were seen as the most common viral families (see Fig. 4a, b). For the largest *Myoviridae*-like phages, unique long whiskers attached to the lower (tail-orientated) part of the capsids were detected. In principle, all phage morphotypes were observed with both TFF and PEG. However, in the latter procedure, a high number of very small *Podoviridae*-like or unclassified phages with capsid sizes smaller than 50 nm were observed (shown on bottom of Fig. 4b) that were not present in the corresponding TFF extraction (Fig. 4a). This result indicates that the PEG methodology might be superior to the TFF procedure with respect to the recovery of small-size phages (putatively not sufficiently retarded by the TFF filtration membrane). For subject Y1 (Fig. 5a, b), different phage types were again detected, one *Myoviridae* phage with long whiskers uniformly attached to the capsids, as well as long capsid-anchored whiskers for a tail-less *Podoviridae*-like phage were identified. Differences in phage population were observed again, as large *Siphoviridae* phages with distinct cross-striations on the tail were only present in the TFF procedure (Fig. 5a) (that apparently could not be collected by PEG precipitation, Fig. 5b). Our detailed TEM analysis confirmed that both TFF and PEG methods are suitable for recovery of intact phages of various morphotypes, as phage ghosts with empty capsids were only observed occasionally. It was furthermore verified that TFF and PEG samples were not contaminated with cells or cellular components (flagella, cell wall debris, etc.).

### Metagenomic analysis of metaviromes

#### Diversity and composition of extracted metaviromes

Phage DNA sequencing libraries of replicates X1 and X2 (both extracted with LIT, PEG, and TFF; see Additional file 1) were prepared using the Illumina Nextera® XT kit and subjected to HTS (MiSeq Illumina platform). The number of pair-ended reads obtained by sequencing of six metaviromes was 43,676,130. After dataset pre-processing (trimming and discarding reads less than 50 nt), 41,180,458 reads remained. Since the number of sequences per metavirome varied between 6,107,696 and 8,457,587, these were subsampled to 5,000,000 sequences pr. virome (mean sequence length of 210 ± 64 nt).

**Fig. 6:**
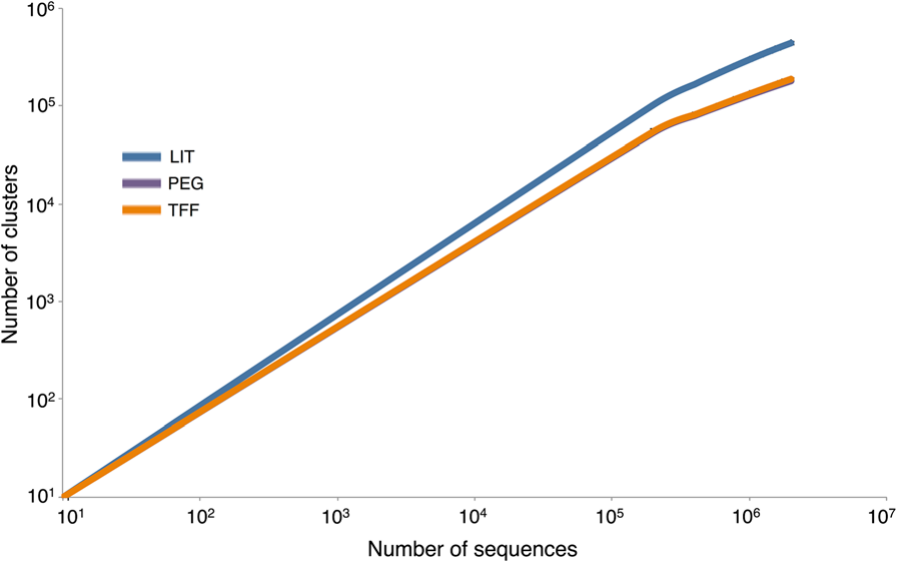
Rarefaction curves estimating the cluster richness for metaviromes derived from LIT, PEG, and TFF protocols. The number of clusters was determined using 2,000,000 reads using a 75 % sequence similarity threshold. The cluster richness of each metavirome was represented by two biological replicates

Variations in the percentage of reads that matched with the NCBI Refseq complete viral genomes protein database (*E* value <10^−3^) were observed. The highest sequence affiliation (known fraction) was found in TFF metavirome (22.6 %), followed by PEG (19.7 %) and LIT (10.7 %). The levels of host and bacterial/eukaryotic DNA in metaviromes based on our optimized protocols were considerably lower (approximately twofold) than what was obtained using LIT (mapped within the unknown fraction or unaffiliated sequences). The percentage of sequences that matched with host DNA in metaviromes extracted with LIT and optimized procedures was >0.3 and 0.1 %, respectively, while for bacterial/eukaryotic DNA (based on mapping against human genome and 16S and 18S rRNA gene databases), >44 and <23 % was determined for LIT and optimized protocols, respectively (Additional file 3). The genetic diversity within the known and unknown fractions (after removing bacterial/eukaryotic and host associated DNA sequences) were screened using alpha-diversity estimates, computed with the UCLUST pipeline [34], and assessed through cluster richness analysis (75 % threshold sequence similarity) [6]. On the rarefaction curves (Fig. 6), similar cluster richness (sequence diversity) was observed between our optimized procedures but slightly lower than LIT. Nevertheless all our observations resembled other phage alpha-diversity that have previously been reported in fecal samples [1].

**Fig. 7:**
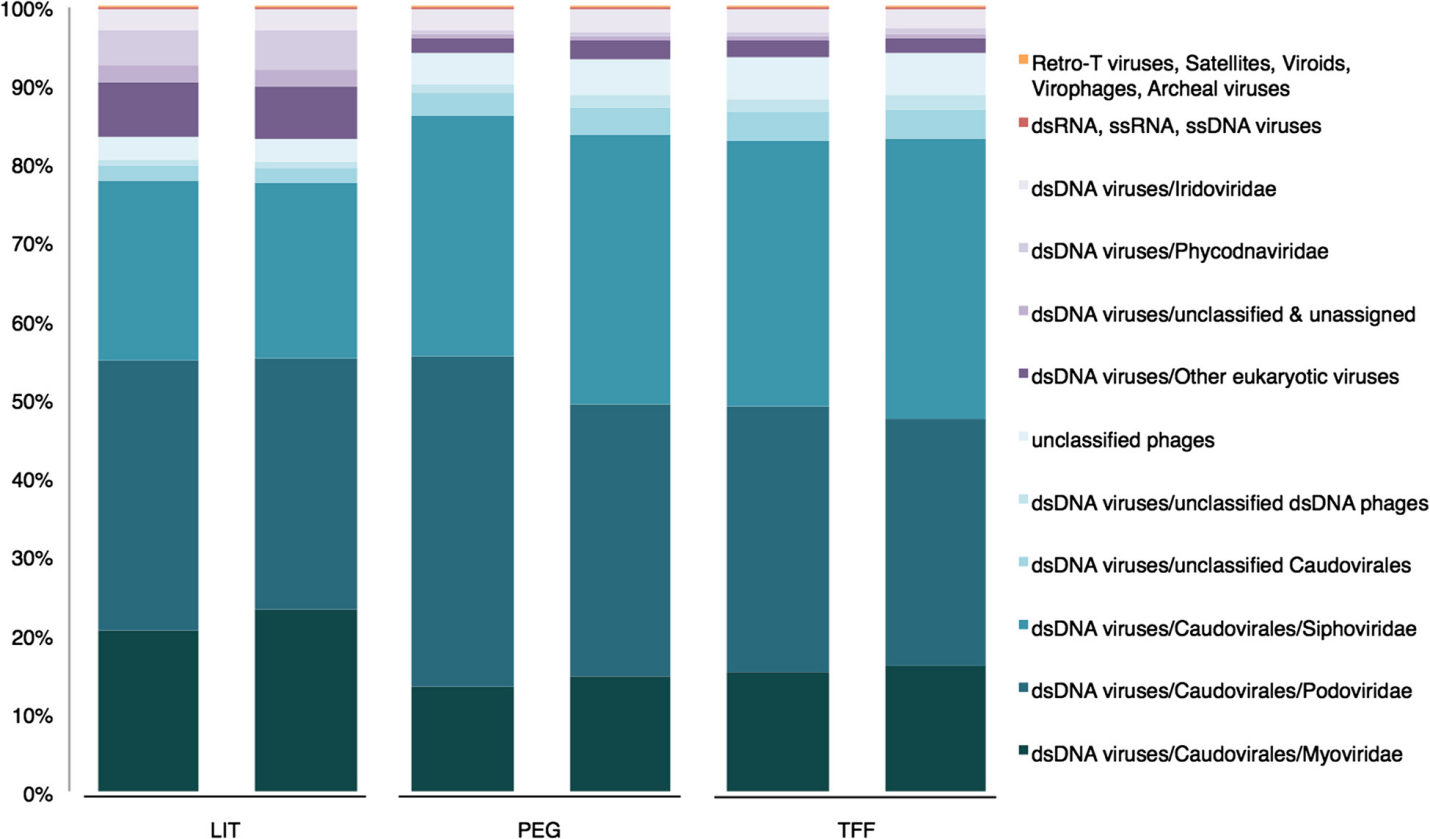
Taxonomic distribution (relative abundance) determined in metaviromes extracted with LIT, PEG, and TFF. The relative distribution is described at the highest taxonomical level (including the most abundant orders and families). Metaviromes reads were annotated using the NCBI Refseq complete viral genomes protein database (*E* value <10^−3^) hosted in the MetaVir web server (http://metavir-meb.univ-bpclermont.fr). The affiliated reads of each metavirome were represented by two biological replicates (samples X1 and X2, see Additional file 1)

Among the known fractions, the composition and abundance at the highest taxonomic level, including the most abundant orders and families, is summarized in Fig. 7. In all metaviromes (using 480,000 reads, accounting for 90 % of the number of affiliated sequences within the most indigent sample), the *Caudovirales* represented the vast majority of the affiliated reads, and these were significantly (*p* value <0.05) higher in PEG (88.2 %) and TFF (86.9 %) compared to LIT (79.6 %). The values are in agreement with results found in studies analyzing the viral diversity of the human gut [11, 12]. We did not attempt to relate the recovery of the spiked bacteriophages to HTS results but rather to evaluate the overall influence of the extraction procedures on the metagenomic dataset. Furthermore, amid the low abundant groups, the proportions of unclassified phages and unclassified dsDNA phages (many of them infecting gut-associated bacteria) were higher (*p*<0.05) in the optimized protocols (>5.6 %) as compared with LIT (4 %). Contrasting with the PEG and TFF, the metaviromes constructed with the LIT protocol had considerable higher relative abundance (*p* <0.05) of non-phage groups (viruses infecting eukaryotes), such as unclassified dsDNA viruses and non-*Caudovirales* groups.

#### *Caudovirales* structure community

The phages of the *Caudovirales* order are generally the most abundant viruses found in the human gut and perhaps one of the most influential entities modulating our GM [35]. Based on their importance, we carried out a separate assessment of the reads belonging only to this viral order and the analysis revealed differences in the distribution of up to 21 bacteriophage subfamilies/genera (Table 1). The LIT procedure (22 %) showed a higher relative abundance of *Myoviridae* compared to the optimized procedures (PEG, 14 % and TFF, 16 %), which was partly given by a substantial increase of unassigned *Myoviridae* and *Peduovirinae* (Table 1). However, the metaviromes extracted with PEG and TFF obtained a larger proportion of the *Siphoviridae* family sequences (>32 %) than LIT (22 %), whose increase was particularly influenced by a boost of unclassified *Siphoviridae* phages.

**Table 1:** Significant changes in distribution of *Caudovirales* subfamilies/genera from metaviromes extracted with LIT, PEG, and TFF

			Relative abundance (%)		
Order	Family	Subfamily/genus	LIT	PEG	TFF
Caudovirales	*Myoviridae*	Bcepmulikevirus	0.05^B^	0.10^AB^	0.12^A^
Caudovirales	*Myoviridae*	Felixounalikevirus	0.09^B^	0.22^AB^	0.32^A^
Caudovirales	*Myoviridae*	Mulikevirus	0.08^B^	0.15^AB^	0.16^A^
Caudovirales	*Myoviridae*	Pbunalikevirus	0.02^A^	0.01^B^	0.01^B^
Caudovirales	*Myoviridae*	Peduovirinae	2.72^A^	0.28^B^	0.28^B^
Caudovirales	*Myoviridae*	PhiCD119likevirus	0.73^B^	1.46^AB^	2.07^A^
Caudovirales	*Myoviridae*	Phikzlikevirus	0.13^A^	0.04^B^	0.05^B^
Caudovirales	*Myoviridae*	Punalikevirus	0.34^A^	0.08^B^	0.07^B^
Caudovirales	*Myoviridae*	*Tevenvirinae*	2.23^A^	1.76^B^	1.78^B^
Caudovirales	*Myoviridae*	Unassigned	3.22^A^	0.63^B^	0.71^B^
Caudovirales	*Podoviridae*	*Autographivirinae*	0.11^B^	0.34^A^	0.36^A^
Caudovirales	*Podoviridae*	Bppunalikevirus	0.10^B^	0.33^AB^	0.37^A^
Caudovirales	*Podoviridae*	Epsilon15likevirus	0.08^B^	0.14^A^	0.14^A^
Caudovirales	*Podoviridae*	Phieco32likevirus	0.10^A^	0.04^B^	0.05^B^
Caudovirales	*Podoviridae*	Unassigned	0.14^B^	0.18^AB^	0.24^A^
Caudovirales	*Podoviridae*	Unclassified	6.16^A^	5.66^AB^	5.21^B^
Caudovirales	*Siphoviridae*	Lambdalikevirus	2.75^A^	0.57^B^	0.71^B^
Caudovirales	*Siphoviridae*	N15likevirus	0.11^A^	0.01^B^	0.01^B^
Caudovirales	*Siphoviridae*	Phic3unalikevirus	0.1^B^	0.15^AB^	0.18^A^
Caudovirales	*Siphoviridae*	Tunalikevirus	0.02^B^	0.13^A^	0.13^A^
Caudovirales	*Siphoviridae*	Unclassified	18.39^B^	30.40^A^	32.91^A^

Values with different superscripts ^(A, B, C)^ were significantly different through one-way ANOVA (using false discovery rate (FDR) correction), which was performed using 1,000 subsampled OTU tables

## Discussion

Recent developments in HTS have generated new opportunities for investigating the diversity and impact of bacteriophages on the bacterial communities in the human gut and on human health as such [1, 17, 36–38]. However, little attention has been oriented towards the efficiency of current protocols dealing with the extraction of bacteriophages. To our knowledge, only one recent study has optimized methodologies to quantify viral particles and to describe their morphological attributes in steps prior to metagenomic studies [26], but the influence of different protocols on the recovery of different phage families through spiking and their overall impact on the phage community structure determined by HTS has not yet been investigated.

Here, we introduced a set of defined, known, and well-characterized bacteriophages (i.e., phages c2, ϕ29, and T4, respectively) that were monitored throughout all extraction steps and were used as representatives of the most common viral families found in the human gut. As our and other studies have demonstrated [9, 31], tangential-flow filtration is indeed a reliable and efficient technique for concentration of viral particles (e.g., 100 kDa filter) but also has limitations when it is employed towards removing large particles other than phages (i.e. microbial cells) as in the case of the 0.45-μm membrane cut-off (possibly due to filter clogging). Our results demonstrated that the recovery rate of the different bacteriophages is very variable and seems highly dependent on the method of extraction chosen. The optimized methods were at least eight times more efficient for c2 and ϕ29 phages and over 286 times more efficient for the T4 phage compared to the reference (LIT) procedure (Fig. 2). The infectivity of T4 (*Myoviridae* representative) was compromised in all protocols during the last step of purification using ultracentrifugation in CsCl gradients. Bourdin et al. [39] showed that various T4-like phages contract their tails and have a tendency to aggregate across the exposed tail tube during ultracentrifugation procedures. Corroborating those observations, qPCR-based quantification of T4 phage DNA showed that this loss in infective T4 phages is not an impediment for further recovery of their DNA as their phage heads are still intact protecting the phage DNA, which could be equally subjected to HTS and metagenome studies.

TFF and PEG protocols extracted between 6 to 10 times more PPs per gram of feces than the LIT protocol. Remarkable interpersonal variation (fecal sample to fecal sample) in the number (Fig. 3) as well as in diversity (e.g. Fig. 4 and Fig. 5) of PPs within each extraction procedure was observed. It can be speculated that this could be an effect of the metabolic state and health status of the individuals donating the fecal sample as these differences have been also observed in bacterial GM structure [40]. DNA yields obtained through LIT did not exceed 3 ng g^−1^ wet sample and are in agreement with yields reported by Thurber et al. [20], from whom the LIT protocol has been adapted. The concentrations of DNA obtained using the reference (LIT) method were much lower than yields achieved with PEG and TFF (see Additional file 2).

The degree of bacterial/eukaryotic and host contamination in the phage DNA was considerably lower in the extractions performed with TFF and PEG. This was accomplished using centrifugation steps (at 5,000×*g*) to remove remaining bacterial cells/host DNA in the phage concentrate, rather than relying on the filtration system performed with 0.45-μm cut-off. Despite our low degree of contamination (in TFF and PEG), we do not rule out the idea that sequences identified as bacterial may have also been derived from active/induced prophages found within our purified PPs what could potentially lead to overestimate bacterial DNA levels. The genetic diversity of every metavirome was explored using cluster richness analysis [6]. None of the rarefaction curves reached a plateau, meaning that the genetic diversity of the metaviromes was not completely covered. Metaviromes derived from PEG and TFF showed similar degree of sequence diversity with each other but slightly lower than the control method (Fig. 6). However, it must be pointed out that the optimization of our protocols was addressed towards recovering a higher yield of bacteriophages, specifically those PPs located in a density fraction ranging from 1.41 to 1.50 g ml^−1^ (see Fig. 1; adjusting the sample density to 1.39 g ml^−1^ allowed us to remove impurities from the phage concentrates). Following these optimized procedures, we increased the proportion of reads belonging particularly to bacteriophages (i.e., *Caudovirales*, unclassified dsDNA phages and unclassified phages, accounting for >92 % of the affiliated sequences) and reduced simultaneously the abundance of sequences associated with eukaryotic viruses (non-bacteriophage groups) (Fig. 7).

Taxonomic assignment and analysis were carried out using raw reads instead of assembled contigs. We chose this approach since contigs may reduce the diversity of the environmental samples, as unique reads of less abundant viruses are difficult to assemble [29]. The percentage of sequences that matched with the NCBI Refseq complete viral genomes protein database was dependent on the extraction protocols: The reads of LIT-based metaviromes had the lowest affiliation (10.7 %), while PEG and TFF had a approximately twofold higher affiliation (19.7 and 22.6 %, respectively). Even though the proportion of known reads for all our metaviromes were similar to those reported in previous investigations [10, 36], the divergence in the percentage of affiliations observed between the adapted and optimized protocols may also have been a response associated with the density fraction recovered from the CsCl gradients (Fig. 1) (i.e., increasing the proportion of non-affiliated reads derived from eukaryotic viruses or non-phage groups).

The metaviromes derived from our optimized protocols yielded a larger proportion of reads classified as *Siphoviridae* (mostly unclassified *siphophages*) but a lower proportion of *Myoviridae* (including *Peduovirinae* and unassigned myophages) as compared with LIT (Table 1). This relative increase of *Siphoviridae* in the metagenomic dataset does not necessarily suggest a true loss of *Myoviridae* phages (as their relative distribution was higher in LIT) but rather indicate an enhanced recovery of the siphophages compared to the LIT method. Unlike the TFF and PEG procedures, in LIT, the 0.45-μm cut-off tangential-flow filtration demonstrated to have poor performance possibly due to filter clogging, thereby triggering a massive loss of PPs what may have negatively influenced the recovery of long-tailed bacteriophages (*Siphoviridae*).

## Conclusion

Here, we report optimized and standardized methods that allow the extraction and recovery of bacteriophages from fecal samples in high yields. Our implemented strategy was focused on minimizing the loss of a spiked set of bacteriophages at the distinct steps of extraction, thereby generating higher yields of DNA prior to library preparation, reducing alterations in the phage DNA extracted and consequently the distribution of phage communities. The directness and robustness of our tested protocols make them suitable for any situation, where dissolution of the solid environmental sample/material is possible. Small adjustment and/or modification of steps, however, may be required according to the nature of the samples of interest.

## Methods

### Samples

Stool samples (anonomyzed) from three subjects were obtained from an intervention trial (3G—gut, grain and greens” carried out at the Department of Nutrition, Exercise and Sports (NEXS), University of Copenhagen. The study protocol was approved by the Ethical Committee of Copenhagen and Frederiksberg Municipalities and performed according to the Helsinki declaration. Stool samples were kept at 4 °C for maximum 24 h after voidance; hereafter, all samples were frozen and stored at −80 °C until use.

### Pre-processing

The strategy for optimization of protocols is described in Fig. 1. Five grams of stool were suspended in 45 ml of sterile SM (sodium chloride magnesium sulphate) buffer (200 mM NaCl, 10 mM MgSO_4_, 50 mM Tris-HCl (pH 7.5), 0.01 % gelatin) and homogenized in filter bags for 2 min at medium speed (Lab Seward, BA7021). As process control, the filtered fraction was spiked with c2 [41], ϕ 29 [42], and T4 [43] phages, each to a concentration of 1.0 × 10^5^ phages g^−1^ feces (*Siphoviridae*, *Podoviridae*, and *Myoviridae* families, respectively). Since these phages could naturally occur in fecal samples, prior to spiking, an aliquot of the filtrated fraction was used to confirm their absence by plaque assays. The concentration of NaCl were left as such (0.2 M contained initially in SM buffer) or adjusted to 0.5 and 1.0 M. Afterwards, the samples were centrifuged at 5,000, 8,000 and 11,000×*g* for 45 min at 4 °C (Fig. 1). The supernatants were recovered and pre-filtrated (dead-end filtration) using glass fiber filters (Millipore, AP2504200).

### PPs enrichment routes

#### Polyethylene glycol precipitation approach (routes 1–5)

Samples were supplemented with 10 % PEG 6,000 (Millipore, 8074915000) [44, 45] and kept at 4 °C overnight after dissolution. PPs were sedimented at 4 °C using two speeds (15,000 and 25,000×*g*) and times (20 and 45 min) of centrifugation as shown in Fig. 1. The obtained pellets (PPs enriched) were re-suspended overnight in 7 ml of SM buffer at 4 °C. To remove low molecular weight contaminants, samples were dialyzed using a 14 kDa MWCO membrane (Sigma, D9277) in 350 ml of SM buffer at room temperature. This step was performed overnight and the SM buffer was exchanged once after 2 h of dialysis.

#### Tangential-flow filtration approach (routes 6–8)

The LIT was adapted from earlier protocols [9, 20, 31] with a slight modification using a larger pore size for micro-filtration [26]. Briefly, samples were filtrated through a 0.45-μm cut-off membrane (Millipore, PXHVMPC50) and the retentate, holding residual cells and micro-particles, was continuously cycled in order to collect the maximum volume of permeate containing the PPs. The 0.45-μm permeates (LIT protocol; route 6) and also pre-filtrated samples (those from the TFF protocol; route 8) were passed through a 100-kDa MWCO membrane (Millipore, PXC100C50) to concentrate PPs and remove low molecular weight residuals. Unlike the former membrane, the PPs were concentrated in the retentate and therefore, its volume reduced down to 7 ml.

### Purification of PPs with CsCl gradients

The purification of PPs using a four-layer CsCl gradient was performed according to procedures earlier suggested [9, 20, 31]. Samples were adjusted to a density of 1.15 g ml^−1^ in a final volume of 10 ml and loaded on top of a 6-ml step gradient, containing 2 ml of 1.35, 1.50, and 1.70 g ml^−1^ CsCl, respectively. These gradients were centrifuged at 82,000×*g* for 2 h at 4 °C, and the fractions between 1.36 to 1.50 g ml^−1^ were collected. For the two-layer CsCl gradient (optimized procedures), samples were brought up to a final volume of 14 ml. Their density adjusted to 1.39 g ml^−1^, loaded on top of 2 ml 1.7 g ml^−1^ CsCl, and centrifuged at 150,000×*g* for 4.5 h at 4 °C. Unlike the four-layer gradient, here, the density fraction comprised between 1.41 and 1.50 g ml^−1^ was recovered. All collected fractions were placed in sterile containers and stored at 4 °C until further use.

### Epifluorescence and transmission electron microscopy

Epifluorescence microscopy was performed following procedures previously described [20, 33]. A small fraction of the phage concentrate (collected from CsCl gradients) was stained with 250× (final concentration) SYBR Gold solution (Invitrogen, S-11494), and PPs were visualized (100× Zeiss Plan-Neofluar) under blue excitation (~495 nm). PPs concentration was estimated using three images (Photometrics CoolSNAP cf) per analyzed sample. For phage quantification, pictures were processed with ImageJ 1.46 using similar parameters of analysis (particle analysis: size, 10–infinity; circularity, 0–1). Negative staining of samples with 2 % (*w*/*v*) uranyl acetate and subsequent TEM analyses were carried out as recently described by Hoyles et al. [26] with a Tecnai 10 transmission electron microscope (FEI Company, Eindhoven, the Netherlands) and a Megaview G2 CCD camera (Olympus SIS, Münster, Germany).

### Extraction of phage DNA

The collected CsCl fractions were dialyzed overnight in autoclaved MilliQ water as described above and then centrifuged in Amicon Ultra filters (Millipore UFC203024) for 15 min at 4,000×*g* at 15 °C. The volume of each sample was then brought up to 450 μl (using sterile MilliQ water), and 50 μl of 10× DNase I buffer were added to a reach a final volume of 500 μl. To remove external DNA, samples were treated with 2.5 U ml^−1^ of DNase I and incubated at 37 °C for 1 h. The extraction of virions was carried out as described by Thurber et al. [20], 0.1 volumes of 2 M Tris HCl/0.2 M EDTA, 5 μl of 0.5 M EDTA (used for inactivation of DNase I), and 1 volume of formamide were added to each sample and incubated at room temperature for 30 min. DNA was spun down by adding 1 volume of 99.9 % ethanol and centrifuging at 14,000×*g* for 20 min at 4 °C; the pellet was washed twice using 70 % ethanol and re-suspended overnight in 537 μl of TE buffer at 4 °C. Subsequently, 30 μl of 10 % SDS and 3 μl of 20 mg ml^−1^ proteinase K were added and incubated for 1 h at 55 °C. DNA was washed and concentrated using DNA Clean & Concentrator (Zymo Research D4010) and eluted in 15 μl of elution buffer (the DNA binding buffer to sample ratio was 1:2), and the DNA concentrations were measured using Qubit® (dsDNA HS assay, Invitrogen).

### Phage recovery efficiency

c2, ϕ29, and T4 phages (*Siphoviridae*, *Podoviridae*, and *Myoviridae* families, respectively) were quantified by plaque assay (top layer 0.4 % agarose; bottom layer 1.5 % agar) using *Lactococcus lactis* subsp. *cremoris* MG1363, *Bacillus subtilis* DSM 547, and *Escherichia coli* DSM613 as host strains, respectively. c2 titers were determined on M17 (5 g l^−1^ glucose (Merck, Germany)), ϕ29 on tryptic soy agar (TSA (Sigma-Aldrich, USA)), and T4 on LB media (per liter, 10 g peptone, 5-g yeast extract, 10 g NaCl, pH 7.2). All substrates were supplemented with 10-mM CaCl_2_ (Merck, Germany) and 5-mM MgCl_2_ (Merck, Germany). Phages (100 μl of serial dilutions) were mixed with host strains in the melted top layer (48 °C). Plates were incubated for 16–18 h at 28 °C for M17 and 37 °C for TSA and LB.

### PP-associated DNA sequencing and data pre-processing

Libraries for DNA sequencing were prepared using the Illumina Nextera® XT DNA kit (San Diego, USA) following the manufacturer’s instructions. Library pooling and normalization were based on the product concentration of the final libraries determined by Qubit® (dsDNA HS). Tagged libraries were sequenced as part of a flow cell of 2 × 300 bp pair-ended MiSeq (Illumina, CA) sequencing. The raw reads were trimmed using CLC Genomic Workbench (CLC bio, Århus, DK, version 7.0.4) using a quality limit of 0.05, allowing zero nucleotides ambiguity. Subsequent analyses were carried out using pair-ended reads and sequences less than 50 nt in length were discarded.

### Analysis of the metagenomic dataset

The composition of the extracted metaviromes was determined using raw paired-end reads using the MetaVir web server (http://metavir-meb.univ-bpclermont.fr; [46]). Since Metavir has limitations analyzing metaviromes containing more than 2,500,000 sequences, the non-assembled datasets (metavirome) were first adjusted to 5,000,000 reads and then split in two sections (2,500,000 sequences each). Composition and relative abundance generated by Metavir were converted into operational taxonomic units (OTU) tables, and subsequent analyses were performed in QIIME (1.7.0 and 1.8.0) [47] . Metaviromes were subsampled using 90 % of the number of sequences within the most indigent sample (multiple rarefactions computed with 1,000 OTU tables). The differences in the relative abundance of phage-taxa (dependent variable) given by paired-comparison between extraction procedures (independent variable) were assessed using one-way ANOVA. The Kraken program [48] was used to map host (human genome NCBI release 71) and bacterial (Kraken Minidatabase, as of 01.08.2015) derived DNA. To get better estimations of uncultured bacteria/eukaryotes, which may not be represented in the previous database, the unclassified reads were also blasted (UBLAST algorithm [34], using -evalue 1e-3, -id 0.7, -query_cov 0.6), against representative sequences of 16S (GreenGenes [49]) and 18S rRNA (Silva [50]) gene databases. Cluster richness analysis [6] was carried out with the UCLUST pipeline [34] using a 75 % sequence similarity threshold (2,000,000 reads were used in each metavirome). Rarefactions curves were computed using 10 subsampled cluster tables and expressed as number of clusters for each metavirome.

## Availability of supporting data

All metaviromes are available through the European Nucleotide Archive (ENA) under the accession number ENA: PRJEB8354 in http://www.ebi.ac.uk/ena/data/view/PRJEB8354.

## Additional files

Additional file 1:
**Description of samples (per subject) used for evaluation of the optimized routes for pre-processing and purification of fecal bacteriophages for virome characterization.** The first biological replicates/extractions are represented by X1, Y1 and Z1, while the second one by *X*2, Y2 and Z2 (each of them were extracted with LIT, PEG and TFF for a total of 18 extractions). All samples and replicates were used for analysis of spiked phage recovery (c2, ϕ29 and T4 phages) and quantification of PPs/DNA. TEM analyses were only carried out with replicates obtained from extractions X1 and Y1 (carried out with LIT, PEG and TFF). The construction of metaviromes was performed using the bacteriophages obtained from Subject X and extracted with LIT, PEG and TFF. (874 KB) Additional file 2:
**Summary of DNA concentrations obtained from the PPs extracted with optimized and adapted methods.** The concentrations of DNA were determined by fluorometry. Values shown represent the measurements of three fecal samples using two biological replicates. (1.08 MB) Additional file 3:
**Metaviromes reads associated with human and bacterial genomes.** Percentage of reads are based on 5,000,000 sequences per phage metavirome. (55.7 KB)

## Abbreviations

GM: gut microbiome
HTS: high-throughput sequencing
LIT: literature-adapted protocol
PEG: polyethylene glycol precipitation
PPs: phage particles
TEM: transmission electron microscopy
TFF: tangential-flow filtration
