# Supplementary material for: Optimizing protocols for extraction of bacteriophages prior to metagenomic analyses of phage communities in the human gut
Source: Microbiome. 2015 Nov 17;3:64. doi: 10.1186/s40168-015-0131-4 (PMC4650499; doi:10.1186/s40168-015-0131-4)
Supplement: Additional file 1: — Description of samples (per subject) used for evaluation of the optimized routes for pre-processing and purification of fecal bacteriophages for virome characterization. The first biological replicates/extractions are represented by X1, Y1 and Z1, while the second one by X2, Y2 and Z2 (each of them were extracted with LIT, PEG and TFF for a total of 18 extractions). All samples and replicates were used for analysis of spiked phage recovery (c2, ϕ29 and T4 phages) and quantification of PPs/DNA. TEM analyses were only carried out with replicates obtained from extractions X1 and Y1 (carried out with LIT, PEG and TFF). The construction of metaviromes was performed using the bacteriophages obtained from Subject X and extracted with LIT, PEG and TFF. (874 KB) [file 40168_2015_131_MOESM1_ESM.pdf]

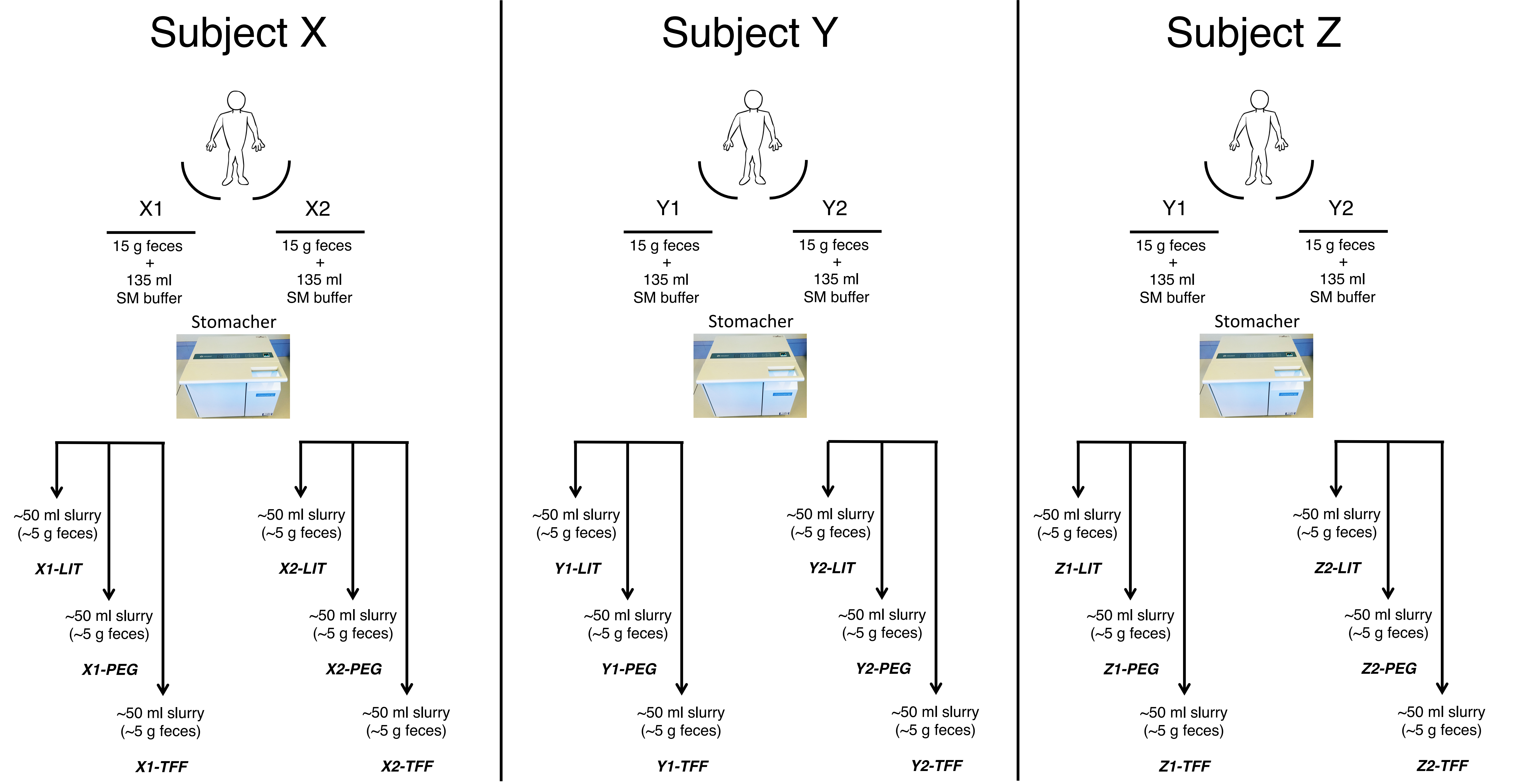

Additional File 1. Description of samples (per subject) used for evaluation of the optimized routes for pre-processing and purification of fecal bacteriophages for virome characterization.

The first biological replicates/extractions are represented by X1, Y1 and Z1, while the second one by X2, Y2 and Z2 (each of them were extracted with LIT, PEG and TFF for a total of 18 extractions). All samples and replicates were used for analysis of phage recovery (c2, ϕ29 and T4 phages) and quantification of PPs/DNA. TEM analyses were only carried out with replicates obtained from extractions X1 and Y1 (carried out with LIT, PEG and TFF). The construction of metaviromes was performed using the bacteriophages obtained from Subject X and extracted with LIT, PEG and TFF.
