# Supplementary material for: Optimizing protocols for extraction of bacteriophages prior to metagenomic analyses of phage communities in the human gut
Source: Microbiome. 2015 Nov 17;3:64. doi: 10.1186/s40168-015-0131-4 (PMC4650499; doi:10.1186/s40168-015-0131-4)
Supplement: Additional file 2: — Summary of DNA concentrations obtained from the PPs extracted with optimized and adapted methods. The concentrations of DNA were determined by fluorometry. Values shown represent the measurements of three fecal samples using two biological replicates. (1.08 MB) [file 40168_2015_131_MOESM2_ESM.pdf]

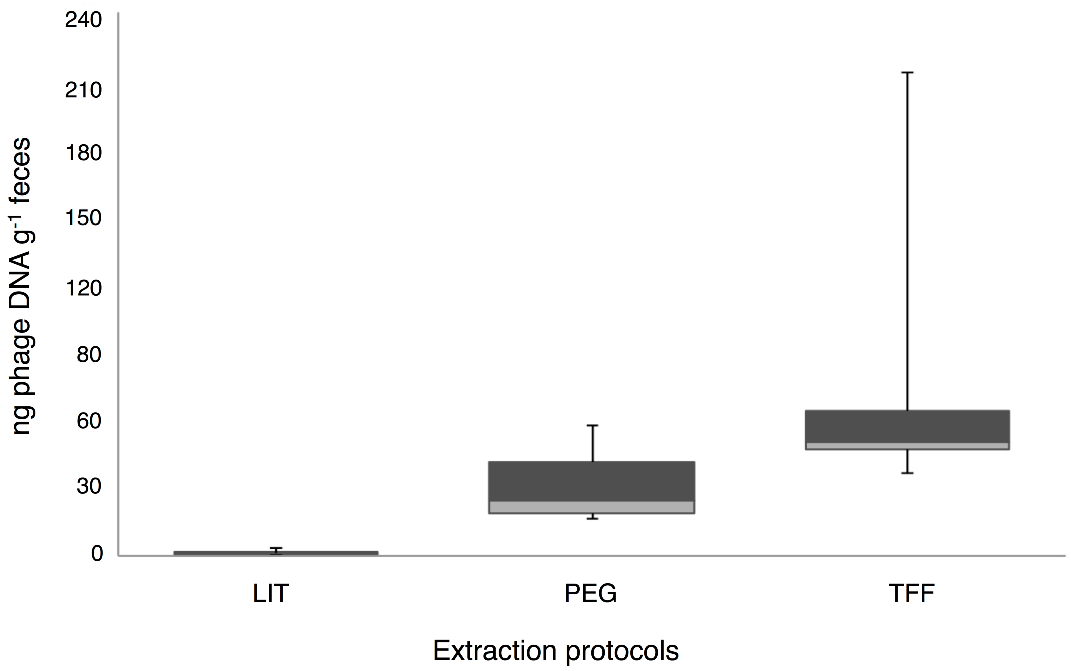

Additional File 2. Summary of DNA concentrations obtained from the PPs extracted with optimized and adapted methods.

The concentrations of DNA were determined by fluorometry. Values shown represent the measurements of three fecal samples using two biological replicates.
