# Supplementary material for: Optimizing protocols for extraction of bacteriophages prior to metagenomic analyses of phage communities in the human gut
Source: Microbiome. 2015 Nov 17;3:64. doi: 10.1186/s40168-015-0131-4 (PMC4650499; doi:10.1186/s40168-015-0131-4)
Supplement: Additional file 3: — Metaviromes reads associated with human and bacterial genomes. Percentage of reads are based on 5,000,000 sequences per phage metavirome. (55.7 KB) [file 40168_2015_131_MOESM3_ESM.pdf]

Additional File 3. Metaviromes reads associated with human and bacterial genomes.

Percentage of reads are based on 5,000,000 sequences per phage metavirome.

	Phage metaviromes' sequences classified as			
Extraction	Human genome	Bacterial genomes	18S rRNA	16S rRNA
LIT_1	0.10%	44.57%	0.002%	0.005%
LIT_2	0.03%	51.34%	0.002%	0.004%
PEG_1	0.01%	22.04%	0.000%	0.001%
PEG_2	0.01%	20.20%	0.000%	0.001%
TFF_1	0.01%	15.02%	0.000%	0.001%
TFF_2	0.01%	18.65%	0.001%	0.001%
